# Primary central nervous system lymphoma—current standards and recent developments

**DOI:** 10.1093/nop/npag016

**Published:** 2026-02-23

**Authors:** Matthijs Van Der Meulen, Joost S P Vermaat, Norbert Galldiks, Samar Issa, Jeroen De Bresser, Jeanette K Doorduijn, Jacoline E C Bromberg

**Affiliations:** Department of Neurology, Leiden University Medical Center, Leiden, the Netherlands; Department of Hematology, Leiden University Medical Center, Leiden, the Netherlands; Department of Neurology, University Hospital Cologne, Cologne, Germany; Institute of Neuroscience and Medicine (INM-3), Research Center Juelich, Juelich, Germany; Department of Hematology, Middlemore Hospital, Auckland, New Zealand; Department of Radiology, Leiden University Medical Center, Leiden, the Netherlands; Department of hematology, Erasmus MC Cancer Institute, University Medical Center Rotterdam, Rotterdam, the Netherlands; Department of neurology, Erasmus MC Cancer Institute, Brain Tumor Center, University Medical Center Rotterdam, Rotterdam, the Netherlands

**Keywords:** Imaging|, liquid biopsy, MRI, [^18^F]FDG-PET, treatment

## Abstract

Primary central nervous system lymphoma (PCNSL) is a rare aggressive B-cell non-Hodgkin lymphoma confined to the central nervous system, without systemic involvement. The incidence has increased over the past 3 decades. The prognosis has improved in patients up to 70 years old, and this type of lymphoma can be potentially cured. The gold standard of diagnosing PCNSL is histological, usually on a brain biopsy specimen via a neurosurgical procedure. Recent developments in both imaging and laboratory analyses of the cerebrospinal fluid, can be helpful in narrowing the differential diagnosis, diagnosing PCNSL itself, and in follow-up after treatment. This narrative review gives an overview of the epidemiology, diagnosis, and treatment of PCNSL, with an emphasis on recent developments in diagnostic techniques and treatment.

Key points

- The diagnosis of a PCNSL requires a brain biopsy or unequivocal monoclonal B-cells in the CSF
- An [^18^F]FDG-PET scan might be used for diagnostic and prognostic purposes
- HD-MTX remains standard of care, CAR T-cell therapy is a promising new treatment

Primary central nervous system lymphoma (PCNSL) is an extra nodal diffuse large B-cell non-Hodgkin lymphoma confined to the brain, leptomeninges, spinal cord, or the eyes without systemic disease activity.^1^ In the most recent WHO-HAEM5 classification PCNSL is included in the new umbrella group large B-cell lymphomas of immune privileged sites. PCNSL is rare since it accounts for only 2-3% of all primary CNS tumours and 4%-6% of extranodal lymphoma, and only 1% of all lymphomas.^2^^,^^3^ The disease is slightly more prevalent in men than in women (1:1.2).^4^ The incidence of PCNSL has increased over the last decades, from 0.30 per 100.000 to 0.44-0.47 per 100.000, but this increase was seen mainly among those >60-years old. With the introduction of highly active antiretroviral therapy (HAART), PCNSL has become very rare among HIV-patients. The increase in incidence is mainly seen among immunocompetent patients. Although this pattern has been described in multiple population-based studies, it is still not fully understood why this increased incidence has occurred.^5^^,^^6^

The median age at presentation is 65-67 years-old.^2^^,^^5^  The most frequent presenting symptoms are focal neurological deficits (70%), headache (51%), and cognitive disturbances/behavioral changes (43%), and seizures (14%).^7^ Due to the aggressive nature of a PCNSL, symptoms develop in days to weeks in most patients. With the introduction of better therapy, that will be described later, the survival has increased, but this improved prognosis is limited to those <70-years old.^5^ In general, the 5-year overall survival (OS) increased from 20 to 40% in the total PCNSL population and from 30 to 60% in those under the age of 60 years.^5^

When PCNSL is suspected, based on brain magnetic resonance imaging (MRI) appearance, it is important to screen for other disease localizations; currently a [^18^F]fluoro-2-deoxy-D-glucose (FDG)-positron emission tomography (PET) scan is considered standard of care to rule out concomitant systemic lymphoma. Ophthalmological examination is also advised with fundoscopy and slit lamp examination to rule out vitreoretinal involvement. Only 4% of patients report ocular symptoms, whereas 15%-20% of patients are reported to have asymptomatic ocular involvement.^8^^,^^9^ A lumbar puncture to analyze the cerebrospinal fluid for lymphoma cells can be done for both staging and diagnostic purposes (as described later on).

Known prognostic factors are age and Karnofsky Performance Score (KPS), with higher age (>50 years old, or >60 years old, depending on the model used) and KPS <70 being independent factors associated with a worse prognosis. Moreover, an elevated serum lactate dehydrogenase (LDH), elevated protein level in the cerebrospinal fluid (CSF), and the involvement of deep brain structures (ie, brain stem, cerebellum, basal ganglia, and periventricular) are associated with a worse prognosis.^10^^,^^11^

This review aims to provide an overview of the diagnostic techniques, and the developments in diagnosing and treating patients with a PCNSL.

## Diagnosing

### Imaging with CT/MRI

**Figure 1.**
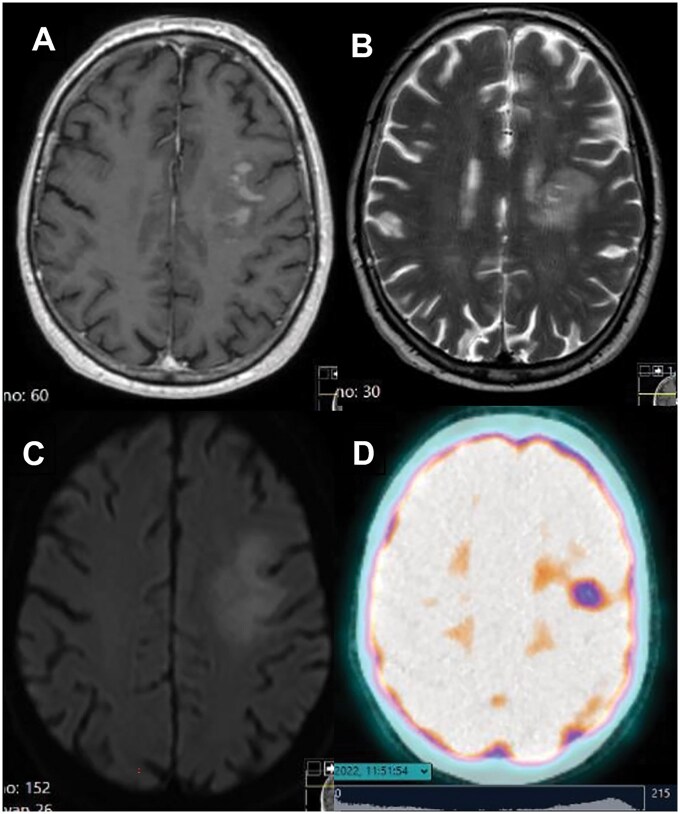
Multifocal primary central nervous system lymphoma in the left frontal and parietal lobe with (A) homogenous enhancement, (B) a low T2 signal, (C), diffusion restriction, and (D) FDG avid on the [^18^F]FDG-PET scan.

On a Computed Tomography (CT), the lesion is typically slightly hyperdense on non-contrast-enhanced series (which is quite typical for PCNSL) and avidly enhances after contrast administration. The majority of patients (65%) present with a solitary space occupying contrast-enhancing intra-parenchymatous lesion. Multifocal disease occurs in 35% of patients and incidentally a PCNSL can be seen as leptomeningeal contrast-enhancement only. The lesion or lesions are most frequently located periventricularly, in the corpus callosum, or in the basal ganglia. Typically, PCNSLs are characterized by: (Figure 1A-C) iso- to hypointensity on T2-weighted images, diffusion restriction, and a homogenous pattern of contrast-enhancement on T1-weighted images after administration of gadolinium based contrast. Intralesional hemorrhage and calcifications are rare.^12^^,^^13^ Diffusion restriction is seen with a relatively low apparent diffusion coefficient (ADC) values (typically with minimum ADC values between 400 and 600 × 10^-6^ mm^2^/s).^14^ This can be used to distinguish glioblastoma and metastasis from PCNSL, ie, in PCNSL, ADC values are generally considerably lower.^15^ Although most lesions are enhancing lesions, rarely, non-enhancing space-occupying lesions occur, sometimes in addition to the enhancing lesions. These lesions may become smaller or even resolve after treatment.^16^ In immunocompromised patients the typical MRI features may be absent and a PCNSL can have patchy enhancement or ring enhancement.^17^ An MRI spine including contrast admission is recommended when there are symptoms localized in the spine.^18^

On MRI perfusion imaging, there is increased perfusion in PCNSL. Although many other brain tumors also have increased perfusion, some differences exist. Using perfusion-weighted MR imaging (PWI), cerebral blood volumes in patients with PCNSL are usually lower than in patients with glioblastoma, measured with dynamic susceptibility contrast (DSC).^19^ When using dynamic contrast-enhanced (DCE) PWI, there is no significant difference between PCNSL and glioblastoma.^20^ The relative cerebral blood volume (rCBV), measured with PWI is also more accurate with DSC than with DCE.^21^ However, there is a strong correlation between K_trans_ (measured with DCE) and relative cerebral blood flow (relCBF, measured with DSC), *P* < .001).^22^

MR spectroscopy might also be helpful for diagnostic purposes and in PCNSL this is usually characterized by large choline peaks, high choline/creatinine ratio, decreased NAA and also a lactate peak.^23^^,^^24^

Other MR sequences such as susceptibility-weighted MR imaging (SWI), highly sensitive for imaging of of small veins, blood and its products, and calcifications, appear valuable in differentiating PCNSL from other contrast-enhancing brain tumors.^25^ For instance, microhemorrhages and calcifications are rare in PCNSL, whereas these findings are frequently observed in malignant gliomas.

Although these techniques are helpful in making a radiological suggestion of the diagnosis, a neurosurgically obtained tumor specimen remains necessary in most cases, considering the wide differential diagnosis, including other brain tumors (eg, glioma, brain metastases), infectious diseases (eg, cerebral abscess, toxoplasmosis, or tuberculosis), and inflammatory diseases, such as tumefactive multiple sclerosis, sarcoidosis, and auto-immune encephalitis. Clinical symptoms and radiological appearances can help to narrow down the differential diagnosis.^26^

### Imaging with [^18^F]FDG-PET

A [^18^F]FDG-PET scan is the advised method to screen for systemic disease involvement when a PCNSL is suspected. This technique has a higher sensitivity and specificity compared to a chest/abdomen CT.^27^ It is important to distinguish a PCNSL from a systemic lymphoma with central nervous system involvement (secondary CNS lymphoma), since the treatment is different.

PET imaging can also show abnormal uptake in the brain lesion in case of a PCNSL. Due to the high uptake of glucose by healthy brain tissue on a [^18^F]FDG-PET scan, it can be difficult to distinguish healthy brain tissue from a lymphoma lesion. However, a small retrospective study showed that 83% of PCNSL patients had a [^18^F]FDG avid lesion on the PET scan, corresponding with the MRI lesion. The [^18^F]FDG-PET avidity was more often present when there were typical MRI lesions (90%). When detected, PCNSL before treatment initiation can have a 2-5 times higher glucose uptake compared to the healthy appearing brain tissue, see Figure 1D.^28^

Several studies (range of included patients 46-53) studies suggested that various [^18^F]FDG-PET metrics can be of prognostic relevance: a high standardized uptake value (SUV)_max_ was correlated with a shorter progression free survival (PFS).^28–30^ Another case series showed that a high interim highest tumor-to-normal ratio (hTNR, ie, the uptake in the lesion versus healthy brain tissue) was an independent prognostic factor for a short PFS, with a Hazard Ratio of 9.76 (95% confidence interval 1.9-50.11). In addition, patients with a low interim hTNR_max_ (≤1) had a significantly longer PFS than those with a high interim hTNR_max_(25 versus 36 months).^31^ This low interim hTNR_max,_ suggests a profound response to first-line treatment, which makes the glucose uptake in the lesions more comparable to the healthy brain tissue. A recent large series of 110 consecutive PCNSL patients showed that patients with a high baseline (>17 cm^3^) total metabolic tumor volume (TMTV) had a significantly shorter OS (12.5 versus 74 months). Post-treatment metabolic response also significantly predicts PFS and OS. In this series the MRI response, according to response criteria^32^ was not of prognostic value for either PFS or OS.^33^

During follow-up, PET may be used as prognosticator by measuring the decrease in glucose uptake, as shown above.^31^ A case-report showed that during routine follow-up of PCNSL patient, there was an observation of an increase of glucose uptake on an [^18^F]FDG-PET scan (1.3 times higher than normal contralateral tissue). Despite the absence of enhancement and negative CSF analysis, histopathological examination confirmed a recurrence of a PCNSL.^34^

In summary, in addition to the value of whole body[^18^F]FDG-PET scan in excluding systemic disease, PET scans may also be used in narrowing the differential diagnosis with other brain tumors, or be used as a prognostic marker. However, most of these studies are relatively small, and false-positive findings on PET scans (compared to MRI scans) have been reported, so caution is advised when they are intended to be used for this purpose.^35^

### Brain Biopsy

In the latest World Health Organization (WHO) classification of Haematolymphoid tumours this rare B-cell lymphoma has been reclassified under the primary large B-cell lymphoma of immune-privileged sites.^1^ Most PCNSL are from the ABC/non-germinal centre cell subtype DLBCL.^36^

Since PCNSLs are very susceptible to steroid apoptosis, the lesion can vanish after the use of corticosteroids. The histological diagnosis may then be hampered or delayed due to the low or absent number of B-cells and only apoptotic debris and reactive lymphocytes might be visible.^37^^,^^38^ So refraining from using steroids in patients with suspected PCNSL until the diagnosis is confirmed is paramount.

A brain biopsy is the gold standard to diagnose a PCNSL. A stereotactic biopsy is the preferred method to get a histological specimen. Surgical resection has a higher mortality and has not been convincingly shown to improve the outcome.^7^^,^^39^

### Cerebrospinal Fluid (CSF) Analysis

Although the gold standard in diagnosing PCNSL remains a histological confirmation,^32^ sometimes the diagnosis can be made, based on the analysis of CSF alone combined with typical imaging characteristics. Cell count, glucose, and protein level in the CSF are not specific, but adding flow cytometry to cytology increases the sensitivity by 2-3 times. However, CSF examination based diagnosis of PCNSL is made in only 30% of the cases.^40^^,^^41^ Since a brain biopsy potentially carries serious risks, such as an intracranial hemorrhage (3.8%) or infection,^42^ finding a diagnosis in a less invasive way would be helpful, especially in frail or older patients. A recent review by a working group of the response assessment in neuro-oncology (RANO) group summarized the role for liquid biopsies in PCNSL.^43^ They have described several biochemical and immunological markers that are increased in the CSF of PCNSL patients, including Interleukin (IL) 6 and 10, chemokine CXCL13, and antithrombin III. However, none are very specific for PCNSL, they can be increased in other brain diseases, including other tumors, and inflammatory brain conditions. Furthermore, none of the markers showed an association with OS.

A potentially valuable molecular marker is the gene myeloid differentiation primary response gene 88 (*MYD88*) that activates the NF-KB pathway, which is usually upregulated in PCNSL. Together with *MYD88*, mutated CD79B is also often found. The most frequent mutation in the *MYD88* gene is L265P, with a frequency up to 88%.^44^ In PCNSL patients, this mutation has been found with a low frequency in plasma (4-57%), but high in CSF (63-92%).^45^ The RANO working group described *MYD88* and IL-10 as potential markers for clinical application, for establishing a diagnosis on CSF examination when a tumor is located very deeply or in frail patients and a biopsy is not safely possible. However, no clinically validated biomarkers other than cytology and flowcytometry for diagnosing and managing PCNSL patients currently exist, due mainly to the small sample size and heterogeneity of patients’ cohorts and the different techniques used across the different studies.^43^^,^^46^

## Treatment

### Induction Therapy

Over the last decades the cornerstone of treatment of PCNSL has been high-dose methotrexate (HD-MTX). The anti-proliferative efficacy is based on the anti-folate mechanism, since folate is used by rapidly dividing malignant cells. Methotrexate is one of the few cytostatic drugs with a high penetration of the blood brain barrier. For induction treatment, HD-MTX is generally combined with other chemotherapeutic agents; a meta-analysis including only prospective trials suggests improved CR rates when adding other agents.^47^ However, no studies have compared regimens directly, except in elderly patients or when adding agents to a basic backbone.^48^^,^^49^ Currently, commonly used regimens are MATRIx,^49^ (R-)MBVP + HD-cytarabine,^50^^,^^51^ MT-R,^52^ and R-MPV.^53^ MATRIx consists of HD-MTX, HD-cytarabine, thiotepa, and rituximab; MBVP consists of HD-MTX, teniposide/etoposide, carmustine, and prednisolone; MT-R of methotrexate, temozolomide, and rituximab, and lastly R-MPV consist of rituximab, methotrexate, vincristine, and procarbazine. Although these regimens have not been compared with each other in a randomized controlled trial, a large retrospective series (n = 346) did not show any difference in survival between MATRIx and (R-)MBVP.^54^ This study also confirmed that the addition of HD-cytarabine (Ara-C) to HD-MTX improves survival, which is in line with a previous randomized trial.^55^ Lastly, there is no additional value for rituximab, when added to MBVP + HD-cytarabine. Combined with other regimens, the additional value of rituximab is uncertain.^56^

### Consolidation Therapy

Without consolidation, the risk of relapse is high. The addition of consolidation therapy, to one of the above described induction therapies improves survival.^54^ There is an ongoing debate about which consolidation therapy is most effective, while preserving neurocognitive functioning and health-related quality of life (HRQoL): autologous stem cell transplantation (ASCT) versus whole-brain radiotherapy (WBRT). Two randomized controlled trials addressed this question.^51^^,^^57^ Both studies used a thiotepa-based myeloablative regimen and showed no difference in OS, one of the studies showed a difference in PFS in favor of ASCT, but this was in only 76 patients.^51^ Both studies, however, showed worse neurocognitive functioning and HRQoL in those who received WBRT (36-40 Gy), compared to those treated with ASCT. However, ASCT is associated with a higher treatment related mortality, of up to 10%. Moreover, ASCT is associated with a higher rate of infections, including aspergillosis and pneumocystosis, and serious (ie, grade 3/4) mucositis.^51^^,^^57^ Both trials included patients with an upper age limit (60 and 70 y, respectively). This seems prudent because of the high toxicity of the thiotepa based conditioning regimens [eg, thiotepa-carmustine (TC) or thiotepa-busulfan-cyclofosfamide (TBC)]. The PRECIS trial included patients up to 60 years. The conditioning regimen used was TBC. The treatment related mortality (TRM) was 11%. The 8-year OS (from upfront randomization) with ASCT was 54%.^51^^,^^58^ The IELSG32 included patients up to 70 years and used carmustine-thiotepa conditioning. The TRM was 3%. The 7-year OS was 56%.^57^^,^^59^ In older adult patients ASCT has been reported with an adapted thiotepa dose (50%) (MARTA trial). The toxicity was still very high, and the TRM 6%. Therefore, a thiotepa based regimen preceding ASCT is recommended, because of the very high efficacy (3-y PFS 75%), although, when treating frail patients, age and patient comorbidities should be taken into consideration before initiating myeloablative chemotherapy followed by ASCT. Non-myeloablative chemotherapy are inferior to myeloablative therapy.^60^ Regarding consolidation therapy, the European guidelines recommend HD-MTX based chemotherapy followed by ASCT.^18^^,^^61^

Low dose WBRT (LD-WBRT, 23.4 Gy) may be an alternative consolidative treatment in patients unfit or unwilling to undergo ASCT. It has been shown to extend PFS (n = 52), compared to a historical group with no consolidation, and in a small (n = 14) subgroup who were followed for 5 years, the cognitive functions were preserved, in comparison to full dose WBRT (36-40 Gy).^62^^,^^63^ Moreover, in a large retrospective series, RD-WBRT (<24 Gy) was associated with improved PFS compared with non-myeloablative therapy.^64^ And in a randomized phase II study patients consolidated with LD-WBRT (23.4 Gy) after induction with R-MPV-A had improved PFS compared with patients not consolidated (Hazard Ratio 0.47, *P* = .007). Furthermore, though there were no significant differences between the groups, cognition appeared better preserved in patients consolidated with LD-WBRT likely as a result of recurrences and/or their treatment.^65^

Standard dose WBRT as a consolidation is not recommended due to the cognitive side effects. However, when patients are unsuitable for chemotherapy, upfront radiation may be a palliative option with a median survival of 8 months.^66^ The cognitive side effects, such as decreased memory and executive functioning, should be discussed explicitly, especially when a survival of more than 6–12 months is expected. A meta-analysis showed that cognitive decline in an older adult population is also caused by the tumor itself, illustrated by the improvement of cognitive functions following induction therapy. However, the combination of chemotherapy and radiation causes cognitive decline and leukoencephalopathy and atrophy.^67^ The risk of neurotoxicity increases with time after treatment but may already arise within 1 year after treatment.

In patients who are not eligible for intensive consolidation therapies, several maintenance therapies have also been studied, as an alternative. These include chemotherapeutic agents, such as HD-MTX,^68^ procarbazine,^69^ Temozolomide, lenalidomide, and Bruton Tyrosine Kinase (BTK) inhibitors, such as ibrutinib. All drugs have been studied in small series (n = 13-37), but appeared to improve PFS and OS, compared to historic series with induction therapy alone.^70^

### Treatment of Older and Frail Patients

The treatment in older and frail patients is challenged by comorbidities, such as reduced renal and/or cardiac function. This can complicate treatment with HD-MTX. Furthermore, older patients are at a higher risk of developing neurotoxicity, including cognitive decline, with accompanying atrophy and white matter hyperintensities on MRI. Lastly, there is a scarcity of specific studies in older adult patients, and the definition of “older adult” differs among studies.

As in younger patients the preferred high-dose MTX protocol is unclear, but in those who were eligible to participate in trials HD-MTX was well tolerated. So, it is advised that older adult patients be treated with the same dose intravenous methotrexate when provided with supportive care and monitoring of the renal function if possible. In case of severe renal dysfunction (ie, eGFR < 50 ml/min) lower dosage MTX is advisable.^69^

### Refractory or Relapsed PCNSL

Refractory PCNSL is defined as a radiological response of less than 50% or persisting CSF localization after induction treatment. Relapse is defined as recurrent CNS lymphoma after CR. Although studies are limited on how to treat refractory or relapsed (r/r) PCNSL, current guidelines give a few clear recommendations. First, given the poor prognosis in this situation, treatment should be weighed against palliative care, considering cognitive performance, clinical condition, and comorbidity. Second, when available, it is recommended to include the patient in a clinical trial to enhance knowledge on how to treat r/r PCNSL. Most trials focus on new treatment options. For example, Bruton Tyrosine Kinase (BTK) inhibitors, such as ibrutinib, zanubrutinib or tirabrutinib. Other trials focus on immune modulating medication, such as lenalidomide, pomalidomide and golcadomide, with or without rituximab, or CAR T-cell treatment (see below). If a trial is not available, it is important to distinguish a late relapse (>1 y after completion of treatment). In case of a durable response on first-line treatment (ie, late relapse) a re-challenge with HD-MTX is recommended as induction therapy and in case of a late chemotherapy based on ifosfamide, carboplatin and or etoposide can be considered.^71^ For consolidation, ASCT is strongly recommended in relatively younger patients (<65-70 y-old), if the patient did not receive previous treatment with ASCT. (Reduced dose) WBRT may be considered as consolidation or as palliative treatment.^18^^,^^61^ There are no trials that compared re-challenge HD-MTX versus newer medications (ie, ibrutinib or lenalidomide).

### Novel Therapeutic Approaches

New developments in the treatment for r/r PCNSL include CAR T-cell therapy. A large series of 100 patients with r/r primary or secondary CNS lymphoma were treated with anti-CD 19 chimeric antigen receptor (CAR) T-cell therapy, but a minority had a PCNSL (16%). The 2-year PFS of all patients was 27% and 2-year OS 37%. The risk of severe cytokine release syndrome (CRS) and severe immune effector cell-associated neurotoxicity (ICANS) was within the range of what can be expected. The symptoms of ICANS included, but are not limited to decreased consciousness, aphasia, dysgraphia. An increased risk of ICANS in patients with CNS disease was not observed: ICANS grade 3-4 was found in 18% of the patients, 2 patients died due to neurotoxicity.^72^^,^^73^

In a small retrospective study (n = 27) WBRT bridging was followed by CAR T-cell therapy, which showed promising results, since the median PFS and OS were not reached.^74^ However, the role of CAR-T in this study is still hard to prove, as all patients received WBRT as bridging, which is an effective treatment.

Altogether, the role of CAR-T cell therapy in R/R PCNSL is still not clear.

Lastly, a few other agents have been studied as a treatment of r/r PCNSL, in small group of patients. Among these agents are pemetrexed (n = 10-38), an antifolate chemotherapeutic agent, check point inhibitor nivolumab (4 patients), and PI3K/mTOR inhibitors, such as temsirolimus (6 patients included). These potential new drugs have been summarized in a recent review.^75^

### Follow-up and Response Evaluation

Regarding follow-up with MRI, in 2005, an international working group defined baseline work-up and evaluation criteria for PCNSL.^32^ Response criteria are based on the extent of enhancement on MRI (measured 2 dimensionally), the presence of lymphoma cells in the CSF and in vitreous fluid, and the use of dexamethasone. The MRI response evaluation defines the following categories: complete response (CR): no signs of abnormal gadolinium-based contrast agent enhancement, complete response unconfirmed (CRu): a small but persistent contrast enhancement abnormality likely related to biopsy or focal hemorrhage, partial response (PR): a reduction of ≥50% of the contrast-enhancing lesion, stable disease (SD): <50% reduction and ≤25% increase of the contrast-enhancing lesion, progressive disease (PD): >25% increase in contrast-enhancing lesion, relapse: a new contrast-enhancing lesion after prior CR or CRu. These response criteria do not take non-enhancing lesions into account. A recent retrospective analysis in 115 patients showed that if there was a response to treatment, the extent of radiological response (CR, CRu, or PR) does not reflect PFS and OS.^76^

## Conclusion

PCNSL is a rare disease, despite an increasing incidence. The prognosis has improved, but only in patients younger than 70 years old. The diagnosis is suspected mainly by using brain MRI, but a definite diagnosis requires a brain biopsy or unequivocal cytological lymphoma and/or monoclonal B-cells in the CSF. Liquid biopsy analysis of the CSF has a potential of making a diagnosis without the need for brain biopsy, but larger validation studies are needed. Induction treatment should consist of a HD-MTX-based combination regimen preferably also utilizing HD-cytarabine. Consolidation treatment is strongly recommended and ASCT is preferred in patients fit enough to undergo this treatment. In r/r PCNSL CAR T-cell therapy is promising, but inclusion in prospective studies is essential to move the field forward.
